# Supplementary material for: Late Acheulian Jaljulia – Early human occupations in the paleo-landscape of the central coastal plain of Israel
Source: PLoS One. 2022 May 11;17(5):e0267672. doi: 10.1371/journal.pone.0267672 (PMC9094563; doi:10.1371/journal.pone.0267672)
Supplement: S1 Table — (DOCX) [file pone.0267672.s008.docx]

| samples | U(ppm) | Er | Th (ppm) | Er | K (%) | Er |
| --- | --- | --- | --- | --- | --- | --- |
| JAL 3 | 2.341 | 0.078 | 4.584 | 0.107 | 0.321 | 0.008 |
| JAL4 | 2.608 | 0.074 | 1.554 | 0.082 | 0.133 | 0.006 |
| JAL5 | 3.978 | 0.093 | 1.327 | 0.091 | 0.082 | 0.007 |
| JAL6 | 2.835 | 0.098 | 4.613 | 0.136 | 0.324 | 0.01 |
| JAL9 | 4.789 | 0.07 | 1.657 | 0.07 | 0.108 | 0.005 |
