# Supplementary material for: Late Acheulian Jaljulia – Early human occupations in the paleo-landscape of the central coastal plain of Israel
Source: PLoS One. 2022 May 11;17(5):e0267672. doi: 10.1371/journal.pone.0267672 (PMC9094563; doi:10.1371/journal.pone.0267672)
Supplement: S2 Table — *IC measurement. (DOCX) [file pone.0267672.s009.docx]

| Si /Al | Ca / Si | *CaCo_3_ % | P % | K % | Mn % | Fe % | Al % | Ca % | Si % | Unit | # |
| --- | --- | --- | --- | --- | --- | --- | --- | --- | --- | --- | --- |
| 3.04 | 0.03 | 0.08 | 0.36 | 0.52 | 1.43 | 4.57 | 9.24 | 0.77 | 28.04 | G-4b | 1 |
| 4.6 | 0.1 | 7.67 | 0.05 | 0.4 | 0.06 | 2.03 | 7.17 | 3.44 | 33 | C-5 | 2 |
| 2.68 | 1.12 | 41.2 | 0.02 | 0.28 | 0.08 | 1.48 | 6.14 | 18.49 | 16.44 | C-4 | 3 |
| 2.67 | 0.14 | 4.75 | 0.96 | 0.74 | 0.12 | 3.18 | 10.12 | 3.73 | 27.01 | D-2 | 4 |
| 2.08 | 2.56 | 69.22 | 0.84 | 0.29 | 0.25 | 1.11 | 4.71 | 25.12 | 9.81 | D-4 calc. | 5 |
| 1.98 | 3.19 | 82.73 | 0.4 | 0.26 | 0.08 | 0.63 | 4.38 | 27.61 | 8.66 | B-1 calc | 6 |
| 5.39 | 0.03 | 0.42 | 0.04 | 0.38 | 0.2 | 2.2 | 6.64 | 1.22 | 35.8 | D-5 | 7 |
| 4.46 | 0.05 | 0.42 | 0.45 | 0.5 | 0.48 | 2.2 | 7.47 | 1.55 | 33.33 | D-4 | 8 |
| 1.93 | 3.37 | 85.74 | 0.01 | 0.2 | 0.03 | 0.58 | 4.36 | 28.31 | 8.41 | A-3 calc | 9 |
| 3.92 | 0.05 | 1.75 | 0.15 | 0.35 | 1.34 | 3.52 | 7.87 | 1.5 | 30.87 | B-4 | 10 |
| 3.81 | 0.05 | 1.5 | 0.43 | 0.36 | 1.03 | 4.9 | 7.7 | 1.4 | 29.37 | G-4d | 11 |
| 1.76 | 5.14 | 72.98 | 0.01 | 0.2 | 0.04 | 0.37 | 3.45 | 31.24 | 6.08 | A-3 calc | 12 |
| 2.87 | 1.26 | 0.5 | 1.81 | 0.84 | 0.23 | 3.09 | 9.49 | 3.43 | 27.19 | B-1 Hamra | 13 |
| 2.96 | 2.12 | 64.47 | 0.01 | 0.27 | 0.16 | 0.75 | 4 | 25.17 | 11.85 | C-4 calc | 14 |
| 4.12 | 0.05 | 2.34 | 0.08 | 0.63 | 0.11 | 2.86 | 7.87 | 1.78 | 32.4 | B-5 | 15 |
