# Supplementary material for: Late Acheulian Jaljulia – Early human occupations in the paleo-landscape of the central coastal plain of Israel
Source: PLoS One. 2022 May 11;17(5):e0267672. doi: 10.1371/journal.pone.0267672 (PMC9094563; doi:10.1371/journal.pone.0267672)
Supplement: S3 Table — *IC measurement. (DOCX) [file pone.0267672.s010.docx]

| Si /Al | Ca / Si | *CaCo_3_ % | P2O5 | K2O | MnO | Fe2O3 | Al2O3 | CaCO3 | SiO2 | Unit | # |
| --- | --- | --- | --- | --- | --- | --- | --- | --- | --- | --- | --- |
| 3.04 | 0.03 | 0.08 | 0.83 | 0.63 | 1.85 | 6.54 | 17.47 | 1.9 | 60.04 | G-4b | 1 |
| 4.6 | 0.1 | 7.67 | 0.11 | 0.48 | 0.08 | 2.90 | 13.55 | 8.6 | 70.66 | C-5 | 2 |
| 2.68 | 1.12 | 41.2 | 0.05 | 0.34 | 0.10 | 2.12 | 11.61 | 46.2 | 35.20 | C-4 | 3 |
| 2.67 | 0.14 | 4.75 | 2.20 | 0.89 | 0.16 | 4.55 | 19.13 | 9.3 | 57.84 | D-2 | 4 |
| 2.08 | 2.56 | 69.22 | 1.93 | 0.35 | 0.32 | 1.59 | 8.90 | 62.7 | 21.01 | D-4 calc. | 5 |
| 1.98 | 3.19 | 82.73 | 0.92 | 0.31 | 0.10 | 0.90 | 8.28 | 68.9 | 18.54 | B-1 calc | 6 |
| 5.39 | 0.03 | 0.42 | 0.09 | 0.46 | 0.26 | 3.15 | 12.55 | 3.0 | 76.66 | D-5 | 7 |
| 4.46 | 0.05 | 0.42 | 1.03 | 0.60 | 0.62 | 3.15 | 14.12 | 3.9 | 71.37 | D-4 | 8 |
| 1.93 | 3.37 | 85.74 | 0.02 | 0.24 | 0.04 | 0.83 | 8.24 | 70.7 | 18.01 | A-3 calc | 9 |
| 3.92 | 0.05 | 1.75 | 0.34 | 0.42 | 1.73 | 5.04 | 14.88 | 3.7 | 66.10 | B-4 | 10 |
| 3.81 | 0.05 | 1.5 | 0.99 | 0.43 | 1.33 | 7.01 | 14.56 | 3.5 | 62.89 | G-4d | 11 |
| 1.76 | 5.14 | 72.98 | 0.02 | 0.24 | 0.05 | 0.53 | 6.52 | 78.0 | 13.02 | A-3 calc | 12 |
| 2.87 | 1.26 | 0.5 | 4.15 | 1.01 | 0.30 | 4.42 | 17.94 | 8.6 | 58.22 | B-1 Hamra | 13 |
| 2.96 | 2.12 | 64.47 | 0.02 | 0.33 | 0.21 | 1.07 | 7.56 | 62.8 | 25.37 | C-4 calc | 14 |
| 4.12 | 0.05 | 2.34 | 0.18 | 0.76 | 0.14 | 4.09 | 14.88 | 4.4 | 69.38 | B-5 | 15 |
