# Supplementary material for: Late Acheulian Jaljulia – Early human occupations in the paleo-landscape of the central coastal plain of Israel
Source: PLoS One. 2022 May 11;17(5):e0267672. doi: 10.1371/journal.pone.0267672 (PMC9094563; doi:10.1371/journal.pone.0267672)
Supplement: Attachment — Submitted filename: Response to reviewrs.docx [file pone.0267672.s011.docx]

Dear Editor,

Thank you for the treatment of our manuscript. We thank you and the three reviewers for your helpful comments and suggestions. We followed your advice and focused our paper on the chronology and geomorphology of the site, while minimizing the behavioral interpretations based on the lithic assemblage. The introduction, discussion and conclusions were revised accordingly.

A few of our figures were revised and updated. Following the comments of Reviewer #3 and due to copyrights inquiries raised by the journal the basemap of Figure 1a was changed, and additional section (1c) was added to the figure, presenting the geological settings of the site and the main streams that relate to our reconstruction of site formation processes. All basemaps used in this figure are open access and referred to. In addition, Figures 13 and 15 were also modified to better present the required data.

Table 9, which was missing from the original submission (our apologies) was added to the description of the lithic assemblage. An additional table, depicting the shape and composition of the bones found on site was added to the Fauna results section (Table 10), as a part of a revision and rephrasing of that section. In addition, in response to a suggestion made by Reviewer #2, we added a table to the discussion, which summarize all absolute dating results from the find-bearing Unit 4, and the overlying Unit 5 (Table 11).

In the process of revision, some of the citations that appeared on the original paper were removed or changed location in the text. Therefore, citation and reference numbering were also modified (not in the TC version).

Following, is a note-to-note response to the received comments. We hope that with these changes you will find that our paper fits the criteria for publication in PloS One.

Sincerely,

Maayan Shemer, on behalf of the authors.

Reviewer #1

1) I consider that the main cope of the article is somehow missing. Is it a presentation of the chronology? A general description of the industry? the general context of the site? I encourage the authors to define clearly from the beginning the aim of it.

Accepted. The main scope of the paper is the chronology, geomorphology, and site formation. Comprehensive lithic analyses of the site are not yet finished and will be discussed in the future. We revised the introduction and the discussion section, removing some of the observations and speculations that were based on the lithic assemblage.

2) Along with the data presentation, the authors constantly include the mention of its assignation to the Late Acheulian without a clear definition of the main features of this period. It is crucial to have a clear understanding of this technocomplex/industrial expression... Perhaps it would be better to define in a sentence what elements can classify an assemblage as Late Acheulian or if the author's criteria are based on chronology, raw material selection, or other aspects.

Accepted. The main attribution of the site to the Late Acheulian is based on the estimated chronology of the site. A sentence was added at the end of the introduction (Lines 127–128): " Preliminary chronological estimations led to the association of the site with the Late Acheulian."

3) When some of these possible features were analyzed¡, the author indicates that in relation to the flaking productions, cores present as an innovative element the control of volume. But bifacial productions also have this volumetric conception. Please provide us with more information about flaking methods perhaps by using their diachritic examples.

The flint analyses section and its related section in the discussion were revised and rephrased. In general, we do note the volumetric conception of bifacial tools production, moreover in relation to the intriguing trait of producing predetermined flakes from discarded handexes, which was identified in several Late Acheulian sites (Rosenberg-Yefet et al. [42] discuss this subject in detail). A short description of the cores' attributes and definitions can be found in the results section of the lithic analysis (Lines 609–631), and we added a citation referring to the works of Rosenberg-Yefet for detailed methodological description.

We accept the general advice of all three reviewers and decided to focus this paper on the chronological and geo-morphological data presented in it. The flint industries are now discussed as extremely preliminary results and used mainly to show similarities to other Late Acheulian sites in the region. As such we are somewhat cautious regarding the level of analysis presented here and await the completion of lithic processing from the rest of the site. These will allow a comprehensive discussion in the technological and typological attributes.

4) What is the meaning of such an amount of debris (56,3%) in the site's context? Please check figure captions and the types used on the tables.

More information is required to fully answer this question. Debris constituting more than 50% of the total artifacts count is not unusual. This category is often used to show the percentage of low-case by-products, and also the degree of breakage (chunk-flake) or possible *in situ* knapping or degree of post-depositional movement (chips). In this representation, debris category also includes micro-flakes, that could in fact be desired products of recycled cores or of cores-on-flake (however they fall under the commonly used 2 cm bar size of chips). At this point, we cannot estimate if this percentage of debris is normal or extraordinary for the site of Jaljulia, as the data from the rest of the excavated localities is not available yet. The meaning of distinct differences between assemblages will have to be studied according to the sub-category in which they will be manifested (i.e., chips, chunks, etc.)

5) In relation to the classification of cores, the authors merely describe them by a simply morphological approach. Usually, the reduction process could define the existence of a real concept in the flaking methods used, and the final expression of core morphologies hide it. Please try to describe them and not just to define "morphologies" are they discoid? multiplatform? levallois?

Please see response to comment #3. We do use technological definitions (discoid, proto-Levallois, single platform, Multiple platform) for core classification. See, for example Table 8.

6) In relation to this, What is the difference between the levallois and protolevallois documented cores? Volumetrically some pieces of figure 9 are levallois.. the existence of discoid and other volume core results must be taken carefully...

Correct, and the distinction can be difficult. The main technological difference concentrates on less strict application of the criteria used for the definition of Levallois cores. For instance, in Proto-Levallois cores, core circumference is not always fully prepared and lateral and distal convexities of the flaking surface are sometimes only roughly designed.

7) When the authors used discoid core categories, were they real "Boeda's" discoid cores? Terradas discoid definition does not analyze final discoid products that are the tricky and key aspects to be analyzed from cores: knifes pseudolevallois flakes and points, quadrangular flakes, etc. By the way, the discoid cores presented in figure 9 seem to be exhausted cores.

The definition of a core as discoid mainly based on Terradas's classification. Admittedly, the definition in this study is mostly based on cores attributes and their associated CTEs. The reason for that is that final discoid products often show affinities to products of prepared cores and to preferential flakes removed from handaxes, and we could not securely differentiate between the methods. Therefore, here all such products were grouped together. A study of these items and the unique distinction for each method is currently under way.

8) This could also happen with the author's "handaxe with preferential flake scars" that are not clearly defined by using a technological reading (order in negatives).

See response to previous comment.

9) For the conclusions, it would be appropriate to establish a general context of the Acheuliand and particularly to the last episodes, in the European context and perhaps also in Asia, to evaluate the relevance of the data provided by this site.

We accepted the general suggestion of focusing this paper on the chronology and geology of the site, believing that indeed our main contribution relies on these aspects. Therefore, we believe that we cannot support a comprehensive comparative analysis with European and Asian Acheulian occurrences.

Reviewer #2

The core of this paper is a presentation of the geology and dating of the Jaljulia site in Israel. There is also some information on the lithics and the minimal bone assemblage presented. I think the paper should be published for the geology and dating. I don't suspect there is more that could be done with the fauna. For the lithics, they note that they are still under study, and what is presented here I would view as extremely preliminary. As such, I think maybe the focus of the paper in the introduction and in much of the discussion and conclusion is a bit too heavy on the lithics. The authors seem to want to put the site into comparison with, for instance, Qesem Cave but it really seems premature given what is presented here.

Accepted. See response to the first comment of Reviewer #1. We fully agree that the strength of this paper is in the chronology and the geological data provided. According to your suggestions we minimized the lithic-based observations both in the introduction and in the discussion.

Further, it seems like one of the main issues will be reconciling the dating of the archaeological horizons. If all of the dates are accepted at face value, it seems that Unit 4 was exposed and occupied for a very long period of time (i.e. across multiple MIS cycles). This seems unlikely, no? So I had a hard time making sense of that.

We do believe that Unit 4 formed and was exposed over a long period of time. Please see detailed responses to the comments of Reviewer #3.

And in terms of site formation processes, the geology seems quite good, but on the archaeology side it is a bit weaker. On the archaeology site there are some general statements about orientations or preservation but no concrete data. Clearly Unit 4 was deposited in a high energy situation. And the faunal analysis argues that the fauna are derived. The lithic analysis says that some of the material might be derived, but argues that most of it is in situ. But the support for the latter is really missing. I think they could be right. The layer could be deposited in high energy but the archaeology not. But this really needs to be shown. I feel like what is really needed is a better site formation study from the lithics and a better effort to resolve the differing dates from different parts of the site from a lithics perspective (i.e. do the lithics from each area all look the same or do they differ in some interesting ways).

We agree. A full comparative analysis of the lithic assemblages from the site is under-way, but unfortunately will not be ready in time for this paper. It will surely provide interesting data regarding differences between excavated localities. We do strongly believe that the artifacts were not deposited in high energy situation, due to very sharp edges, and little evidence of size-sorting. We do consider the possibility of low-energy water coverage of some parts of the site highly probable, which would account for the poor fauna preservation.

I agree with the authors that this site will make a nice additional data point for the late Acheulian (however, the near complete lack of fauna and no evidence of spatial structure mean some significant limits to what can be said of human behavior). I would though reorient the paper a little less around the lithics and questions of AYCC, Late Acheulian, MP etc. given that in this paper they are clearly secondary to the dating and geology.

Accepted. See response to the first comment of Reviewer #1.

In this introduction, the notion that lithics equal people is pretty clear. For me, I don't think it is clear that lithics equal people, but I realize that this is not the prevailing view of those doing Levantine Paleolithic. Still, maybe some effort could be made to discuss the lithics as a component of human groups that occupied the Levant during these various periods. Whether changes are driven by demographic changes (incoming groups) or represent local changes is mostly (entirely?) unclear throughout this whole period.

After considering the reviews, the introduction was revised. Sentences strongly correlating lithic industries with specific human groups were removed, and lithics are now referred to as ‘industries’. We also include a sentence counting some of the possible causes for lithic variability (Line 75–78): " Nevertheless, lithic studies have suggested techno-typological inter-assemblage variability, implying the probable effect of chronology, geography and the nature of the activities performed, on the lithic diversity within Late Acheulian sites (e.g., [1,3,24,36–41])."

Line 109 - So Late Acheulian is a chronological phase or a technological phase?

Corrected. In this work, the definition of the site as Late Acheulian heavily relies on its chronology. Nevertheless, attributes of the lithic industries support this designation, such as the relative abundant production of predetermined flakes from shaped cores or from discarded bifaces. We see the ambiguity in the sentence and corrected it. The sentence now reads " Difficulties in acquiring absolute ages further add to the ambiguity" (Line 98).

Line 126 - Coming back to my comment above about lithics equal people, the discussion in this paragraph is appropriate.

Accepted. This paragraph was revised, some parts were removed, and others were united with the paragraph above, discussing chronology. It now reads: " However, this approach is viewed with caution, as most scholars view an autochthonic cultural continuum between the Late Acheulian and the AYCC. Based on the plethora of evidence available, the AYCC was suggested to have been developed from Late Acheulian industries, while embracing some of its technological hallmarks (e.g., bifaces, lithic recycling) and introducing a set of new innovative technologies and behaviors (e.g., systematic blade production, Quina scraper production, habitual use of fire, cooking etc.; e.g., [56–57]).

Thus, a more comprehensive understanding of the chronology of the Late Acheulian is still to be desired, and the nature of technological and behavioral transformations that took place during the terminal Lower Paleolithic in the Levant deserves special attention." (Lines 113–119).

The manuscript could use a better section numbering system so that the hierarchy is better understood. For instance, there is "dose rates" on line 279 and 316. I know they related to different dating methods, but it could be made more clear by linking them with section numbers.

The manuscript is organized according to PloS One guideline, based on the "sample manuscript body" provided on the website.

Line 331 - 7 is the oldest or youngest? [I see now it must be the youngest - but state it here]

Corrected. This line now reads " The sequence was divided into seven units, presented here from the oldest (Unit 1) to the youngest (Unit 7)" (Lines 308–309).

Table 3 - Is the size of the lithic assemblage all lithics greater than 5mm (the screen size in most cases)? Maybe state this somewhere. Area G is NA for what reason? And G1-G5 are layers? Is this explained somewhere? [I see now on Line 465 - maybe reference to this needs to go earlier]

Corrected. The caption of Table 3 now reads: " Table 3. Excavation details per locality. Lithic assemblages include all artifacts collected, including from sieving using 5 mm mesh."

Area G was subjected to wet sieving, and its processing (picking, sorting, counting) has not been completed yet. Therefore, we cannot provide a sample size for that area.

A short mentioning of horizons G1–G5 was added to the geological description of Unit 4 in Area G (Lines 400–402): " Four archaeological horizons were identified in the gravelly sub-units of Area G, numbered (youngest to oldest) G1–G4. The oldest gravelly horizon was numbered G5, correlating to Sub-unit 4a. Lithic artifacts were scarce in that horizon."

Line 455 - best state of preservation is based on what?

State of preservation was indicated based on the presence of sharp edges, prominent scar ridges, abundance of chips, and horizontal deposition of large artifacts. This explanation was added in Lines 436–440: " The best state of preservation was noted at the northeastern part of the site, in Areas B, D, E (Fig 4, Figs S2, S4, S5) and G (Fig 5, Fig S6), where artifacts mostly presented sharp edges and prominent scar ridges. Average layer thickness and find density, adding up to thousands of flint artifacts, are shown in Table 4. Artifacts in Areas B, D and G areas were mostly found in horizontal orientation and items smaller than 2 cm (chips) were abundant."

Line 457 - No data on orientations?

Orientation was only documented for large artifacts such as bifaces and cores. These presented mostly horizontal orientation (mentioned in Line 439).

Line 458 - No data on ratios of chips to larger artifacts?

Chips constitute 11% of all artifacts in the assemblage. When grouping together also the micro flakes (flakes smaller than 1.5 cm), they constitute ca 15% of the assemblage. This data appears in Table 7.

Line 459 - How do limestone slabs mean that there is little post-depositional movement?

We consider the stone slabs as possible work-surfaces, as some bear percussion marks, and some seem to be shaped. Notably, no marks of transport by high energy fluvial activity on the slabs (rounding of the edges, inner fracturing) were identified on the slabs, as would be expected considering the geological reconstruction of the formation of Unit 4. The slabs are still under study, so we are somewhat cautious about fully ascribing them as work surfaces, but their possible ascription is mentioned in Lines 440–442.

So Unit 1 is older than Unit 7, but horizons G1 and G2 are younger than horizons G3 and G4. And 4a is older than 4e?

Yes. We tried to create a distinction between archaeological horizons and geologic units. Thus, in the geologic numbering high numbers=younger units, while in the archaeological numbering high numbers=older units. Admittedly, the original numbering of the archaeological horizons followed the lines of the geological numbering (i.e., Horizon G1 was the oldest), but we got feedback that it causes confusion as the correlation between horizons and unit is only partial.

Line 470 - No data for this?

Rephrased. Our description of artifacts preservation state and post depositional erosion is based on observation during field work. That include the abundance of heavily patinated items, edge damage, orientation of large artifacts, and the morphology of Unit 4. In the specific case of Area D, faunal remains also indicate post depositional abrasion. These are detailed in Lines 452–458, however the word "evidenced" in Line 452 was changed to "implied", as we agree that more data is required (e.g., comparative analysis of the flint assemblages from all excavated localities):

" In Area D, post-depositional movement of artifacts was implied by their orientation, which often presented an oblique angle, and by the morphology of Unit 4. Faunal remains, only found in this area of the site, show evidence of water activity, manifested as edge rounding and polishing. At the western part of Area D, Unit 4 was composed of a series of moderate rises and depressions, indicating a dynamic fluvial environment at some point during its deposition, and possibly after the human activity in that locality. In the eastern part of Area D, however, the state of preservation was better, resembling that observed in Areas B and G."

Any explanation for why faunal preservation is only in Area D?

We estimate that it could be related to recurrent alternating periods of wet/dry environments in the other localities (perhaps seasonal flooding), but micromorphological analyses are required to make a more educated determination. Also, we must consider that the fauna remains do not relate to the human activity on site, as no cut marks or typical marrow extraction breakage were identified.

Actual data on artifact preservation by area would be nice (and ratio of small to large finds).

We agree. However as work on the other localities is still under-way, this data is not yet available.

I am not a dating expert, but clearly there are a wide ranges of dates here each with fairly wide error ranges. I think a figure summarizing the results would be good.

Accepted. A table summarizing the ages of Units 4 and 5 was added into the discussion section (Table 11).

Line 591 - Why does Area B have a date? Is the dating by area or for the artifact find horizon?

Samples for absolute dating were collected in all localities, according to geological unit (excluding Area A, where the unit was too cemented). They were mostly collected from the archaeological finds bearing Unit 4, and from the overlying sediments of Unit 5. Therefore, most localities can have their own age estimation.

Table 7 - I don't get what modified and non-modified base flakes are. The base is the platform? And how is a non-modified base flake different from a primary element flake? And if the flake has a lip, it goes in its own category exclusive of these other categories? Shaped items is bifaces and also scrapers etc.? I don't know but there is no lithics method in the methods section. Shouldn't there be something about the methods and terms somewhere here? It sort of seems like the definitions come after the results.

The methodology used for lithic analysis here is based on the works of Rosenberg-Yefet et al. [42, 92]. We added references to publications in which a detailed description is provided (Lines 577–578). We tried to balance the preliminary nature of the data presented here regarding the lithics, with the space it takes in the manuscript. As we revised the paper to focus on chronology and geomorphology, we feel that a full methodology chapter on lithics might be too much. However short descriptions are provided when we thought such were needed.

Line 659 - What are retouched flakes that are not scrapers, denticulates or notches?

Corrected in the text (Line 653). The term refers to flakes with partial retouch that does not create a notch.

I am wondering what the significance is of the bone size plot? I don't understand it either (a ternary diagram with percentages for length, width and thickness). Not sure how to interpret this.

Line 699 - I guess this is the point of the size diagram, but I still don't quite get it.

Corrected. The purpose of the diagram is to support the observation that all fragments fit within the same size range and to provide a base for discussion in the eroded stage of the fauna remains. The fauna section was revised, a paragraph and a table were added to clarify the point.

As I reach the end of the paper, I find the summary of the dates. Maybe a figure is not needed as I gather all of the dates are reported on the schematic section? Maybe not?

We accepted the original comment. A table summarizing the ages of Units 4 and 5 was added into the discussion section (Table 11).

Does it really make sense that Unit 4 was exposed over the course of several MIS stages and yet its occupation in each case always represents wetlands/marsh. Seems likely that it represents less time than that.

See response to Reviewer #3.

Line 929 - Says that more data are needed. But then the authors report a number of highly speculative things with no supporting data (e.g. scrapers with scalar retouch, blade production).

Accepted. The paragraph suggesting the existence of affinities between the Late Acheulian of Jaljulia, and the Acheulo-Yabrudian of Qesem Cave was removed from the discussion.

Line 963 - I didn't get that there were three phases of occupation supported by geomorphology. The dates maybe, but I didn't see that in the geomorphology.

Accepted. Indeed, the suggestion regarding three phases of occupation is based on the chronology. Geomorphology shows only two phases, based on the younger fluvial activity in Area G. The sentence was rephrased and now reads: "The possibilty that at least three phases of occupation are represented on-site, supported by absolute ages, provide a unique opportunity to study diachronic changes in material culture within the Late Acheulian"

I would delete the paragraph on Line 971.

Accepted.

Line 985 - Again, I don't see support for these speculations about links to Qesem Cave.

Accepted. The conclusions section was revised, and the suggested continuity based on lithic affinities was removed.

Reviewer #3

1. Remove all discussion of why the correct age is in any way known in advance of dating. There is simply no archaeological or geomorphological support for such a position and it stands in contrast to excellent analytical work. The claim that the OSL ages are problematic due to high De measures requires full substantiation and seems to directly contradict the earlier statement that there is high confidence in the ages-- which moreover include TT-OSL on quartz and IRSL on feldspars, both methods that work for sites much earlier than the one reported on here. I would point to the large number of dating samples as further strengthening the overall confidence of where in time this site is situated. I found the agreement between the ESR and OSL for Area C particularly impressive. The authors need to at least consider the possibility that this site (Unit 4) formed over a long period of time, as appears to be indicated by the age determinations.

Accepted. The results section of the luminescence dating was revised, and the paragraph discussing the possibly problematic ages due to high De measures was removes. The age disclaimer sentence in the abstract was also removed. However, we do believe some caution is required regarding the very young ages of Area G, as they have major potential implications regarding existing paradigms of prehistoric research. While the method has proven itself very well, we feel that further substantiation of these ages is required.

2. The integration of the geology into the coastal geomorphology is very weak. I do not really comprehend why so much space is given to chemical analysis where we know that all fluvial derived sediments have the same source in the neighboring hills.

The chemical and physical analyses were used to prove and define the local immediate environment – aeolian, fluvial, wetland, soil (Hamra or calcic). The integration to the coastal geomorphology was weak and we strengthen it by correlations to sea level and location of coastlines, sand incursion eastward blocking the Yarkon corridor causing the formation of the Jaljulia marsh, the fluvial regime along the earlier paleo-channel, soils formation and the relation to the settlement (Lines 38–43; 738–742; 750–752; 763–768; 784–789; 863–890).

There needs to be much more serious engagement with fluvial processes, as has been done in paleoclimatic reconstructions at Revadim (Malinsky Buller et al. 2011).

At the Revadim site of Malinsky-Buller et al. (2011) the sizes of the channel allowed estimates of the discharges, whereas at our site, the dimensions of the paleochannel were unknown and therefore we could only calculate the velocity and critical shear stress needed to transport the maximum gravel sizes within the fluvial units based on the relationships of Costa (1983) which are more relevant to actual gravel transport. (Lines 767–768; 786–789; 851–856)

One particularly interesting issue is how to interpret periods of incision, indicated here by what appear to be two channels in a unit underlying the site. This could be evidence of high rainfall but it is equally important to consider the effects of sea level on fluvial dynamics.

Unit no 2 is not dated so it is difficult to correlate it to any specific climate. Nevertheless, large amounts and size of gravel transport were correlated to cold dry Heinrich events and the last glacial period by Greenbaum et al. (2014) in the Qishon stream. We assumed similar climate conditions for Jaljulia (Lines 868–875).

What I most wanted to understand was what the authors think was the depositional context for the archaeological occupation.

Based on the chemical and physical analyses we believe that the depositional context of the occupation was a shallow wetland/water body with very low flows and probably high groundwater levels (Lines 790–795; 875–879).

In the generalized cross section it appears that Unit 4 fills a basin that actually cuts into the earlier deposits, are we looking a broad fluvial channel?

Yes, we believe that there is a change in the pattern of the fluvial system from a more confined channels (Unit 2) cutting into the underlying unit 1 to a much wider probably braided channel (Unit 4) where the eastern border is unknown (lines 784–786)

Finally, from the schematic sections there is reason to question whether Unit 4 actually represents a single depositional unit. Could we be looking at a braided stream that evolved over a long period? This would be logically consistent with the dating results.

Yes, we agree that the deposition of unit 4 extended a long time period as confirmed by the OSL dating, specifically the upper finer part with the archaeological occupation. The lower part with its braided pattern and energetic flows was probably too strong for human occupation (Lines 784–786).

From the images it appears that the artifacts accumulated during the deposition of Unit 4, this should be made more clear-- it might be useful to have some vertical data on artifact distribution by spit in the different areas.

The available vertical data indicates higher frequency of the artifacts at the upper, finer parts of unit 4 and therefore we believe that the occupation is related to the upper part of the unit. (Lines 795–796)

Essentially it looks like what might be taking place is a shift from two incising channels (without associated archaeological material) to a braided stream that flowed through the area and is associated with dense archaeological accumulation.

We agree. See response to previous comments.

For assessing the association between the dated sediments and the artifacts is critical as the age determinations might actually record the time of sediment deposition rather than time of initial artifact deposition.

The frequency of the artifacts was the highest at the top of Unit 4. When sampling for dating we made a point of sampling the artifact-containing horizon. Therefore, we believe that it can be related to the time of artifacts deposition. In Area B, a second sample was collected from the lower, gravelly part of Unit 4. This sample could indicate the time of fluvial deposition of Unit 4 gravels.

3. Adding to these problems is the visual presentation of data. Although Figure 1 is useful we also need a drawing of the excavation areas that is easy to key into Figures 2 and 15.

We made some modifications in Figure 15 and added the geological setting of the site to Figure 1. The geological sequence exposed in each area is presented in Figure 2, and their relative location, in Figure 1b. Additional illustrations of each area is presented as supplementary material.

I also did not understand some of the elements of Figure 15, for example how do the authors know that Unit 1 underlies the entire site if it was only reached in the central area?

Unit 1 was reached in four locations, where it is overlain by Units 2 and 4. The unit also appears in the geological map (Fig 1c)

I am assuming that the energy of Unit 4 was responsible for the erosion into the underlying strata but this is not clear.

Clarified in line 782.

I would have been interested to see a map positioning the site relevant to known paleo-drainage systems.

Accepted – see Figure 1c.

This article appears to dismiss the system of kurkar ridges often included in scenarios for the development of the coastal plain, it would be interesting to also include on a map the location of known kurkar ridges.

Accepted – see Figure 1c.

I found there was consistent use of imprecise language not appropriate for a scientific publication. For example, line 176 "using standard methods for prehistoric archaeology in the Levant" , standard for who? This implies that there were no reasonable alternatives where this is not the case. The authors need to justify their method, not appeal to some alleged consensus methodology. Line 125 "this approach is mostly viewed with caution", by who? why? The authors need to ground assertions.

Accepted. These sentences were removed from both the lithic and the geological methods sections. The whole paragraph in question was revised and rephrased, see response to Reviewer #2 for detailed description of the changes made.
